# Beta-Lactam Antibiotic Concentrations and the Acquisition of Multi-Drug Resistant Bacteria in Critically Ill Patients

**DOI:** 10.3390/life15050739

**Published:** 2025-05-02

**Authors:** Anita Farinella, Michele Salvagno, Andrea Minini, Laila Attanasio, Ana Cunha, Marco Menozzi, Andres Saravia, Filipe Amado, Julie Gorham, Maya Hites, Fabio Silvio Taccone, Elisa Gouvêa Bogossian

**Affiliations:** 1 Department of Intensive Care, Hôpital Universitaire de Bruxelles (HUB), Université Libre de Bruxelles, Route de Lennik, 808, 1070 Brussels, Belgium; anfarinella@ismett.edu (A.F.); michele.salvagno@ulb.be (M.S.); aminini@asst-pg23.it (A.M.); laila.attanasio@ausl.bologna.it (L.A.); alcunha.2@gmail.com (A.C.); marc.menoz@gmail.com (M.M.); andres.saravia@quironsalud.es (A.S.); filipe.amado@hospitalmaranhense.com.br (F.A.); julie.gorham@hubruxelles.be (J.G.); fabio.taccone@ulb.be (F.S.T.); 2 Istituto Mediterraneo per i Trapianti e Terapie ad Alta Specializzazione—IRCCS ISMETT, 90127 Palermo, Italy; 3 Clinic of Infectious Diseases, Hôpital Universitaire de Bruxelles (HUB), Université Libre de Bruxelles, Route de Lenik, 808, 1070 Brussels, Belgium; maya.hites@hubruxelles.be

**Keywords:** antimicrobial resistance, multi-drug resistance, therapeutic drug monitoring, beta-lactams

## Abstract

Antimicrobial resistance (AMR) is a worldwide healthcare emergency. Whether insufficient beta-lactam antibiotic concentrations can be associated with AMR emergence remains controversial. This is a retrospective single-center cohort study including patients admitted to the intensive care unit of a tertiary university hospital from 2009 to 2014, who required a broad-spectrum beta-lactam antibiotic and had at least one therapeutic drug monitoring (TDM). Patients were categorized as having inadequate drug levels if the trough concentration (Cmin) fell below the clinical breakpoint for *Pseudomonas aeruginosa*. AMR was defined according to breakpoints recommended by the European Committee on Antimicrobial Susceptibility Testing (EUCAST) using the disk diffusion method. A total of 444 patients (male sex, *n* = 313, 71%; female sex, *n* = 131, 29%; mean age 58 ± 15 years) were enrolled in the study. Patients received piperacillin/tazobactam (*n* = 168), ceftazidime/cefepime (*n* = 58) or meropenem (*n* = 218); among them, 65 (15%) had insufficient drug levels. Nine of these 65 (13.8%) patients with insufficient antibiotic levels acquired at least one pathogen with AMR within 15 days of TDM, when compared to 84/379 (22%) in the other group (OR 0.56 [95%CI 0.27–1.19]; *p* = 0.13). In a multivariable competing-risk analysis including male gender, APACHE score on admission, previous colonization by other MDR bacteria, urinary catheter, central venous catheter, mechanical ventilation, previous hospitalization and previous surgery, insufficient antibiotic levels were not associated with AMR acquisition (sHR 0.84 [95% CI 0.42–1.68]). Similar results were found when a higher threshold was used to define insufficient drug levels (C_min_ < 4 times the clinical breakpoint). In conclusion, insufficient beta-lactam levels were not independently associated with AMR acquisition. Future prospective studies are needed to evaluate better the relationship between low drug levels and antibiotic resistance acquisition.

## 1. Introduction

Antimicrobial resistance (AMR) is a cause of great concern worldwide and is considered a public health emergency, according to the World Health Organization [1]. In 2019, around 5 million deaths were estimated to be associated with bacterial AMR globally, including 1.27 million deaths attributable directly to bacterial AMR [2]. The rate of development of antibiotic resistance is far superior to the rate of development of new therapeutic options [3], so that, as soon as a new drug is used, some micro-organisms will become resistant [4,5,6]. Indeed, antibiotic use is an essential driver of resistance acquisition, even if rationally used [7].

Critically ill patients are highly susceptible to colonization and infection [8], especially those caused by multi-drug resistant (MDR) bacteria, because of their prolonged hospital stay, invasive procedures and multiple courses of antibiotic therapies [9,10]; moreover, these MDR infections are generally associated with higher mortality rates than those caused by susceptible pathogens [8]. Choosing the appropriate antimicrobial (i.e., with bactericidal activity against the pathogen) and the adequate regimen (i.e., dose and frequency of administration) are essential for successful clinical cure [11]; in particular, adequate drug regimens would result in drug concentrations able to kill bacteria and reduce bacterial growth [12]. However, whether this would also minimize the emergence of MDR pathogens remains unclear [13,14].

Beta-lactam antibiotics are the most frequently used antimicrobials in critically ill patients [15], due to their broad-spectrum activity, wide therapeutic index and safety profiles [16]. Beta-lactams are time-dependent agents, whose clinical and microbiological efficacy is related to the amount of time that the unbound drug concentration remains above the minimal inhibitory concentration of the infecting organism [17,18]. During critical illness, an increased distribution volume (i.e., increased capillary permeability and edema, fluid overload, third-spacing; hypoalbuminemia) or drug clearance (i.e., augmented renal clearance or extra-corporeal therapies) may lead to drug underdosing [16,18,19,20].

Therefore, we aimed to assess whether antibiotic underdosing assessed by therapeutic drug monitoring (TDM) is associated with developing MDR pathogens in critically ill patients. We hypothesized that insufficient drug levels of beta-lactams would independently increase the risk of MDR acquisition.

## 2. Materials and Methods

### 2.1. Study Design

We conducted a single-center retrospective cohort study of adult (>18 years old) critically ill patients admitted to the intensive care unit (ICU) of Erasme University Hospital (Brussels, Belgium) from January 2009 to December 2014. This study was performed according to the Declaration of Helsinki and was approved by the local Ethics Committee (P2018/495); the need for informed consent was waived due to the retrospective design. Inclusion criteria were as follows: age > 18; ICU length of stay of at least 7 days; presence of an infection on admission or development of infection during the ICU stay; use of a broad-spectrum beta-lactam antibiotic to treat the infection (i.e., cefepime or ceftazidime, CEF; meropenem, MEM; piperacillin/tazobactam, TZP) with at least one TDM measurement available. The only exclusion criterion was antibiotic prophylaxis administration for no more than 48 h before the beginning of beta-lactam treatment for an infection. This study followed the recommendations of the Strengthening the reporting of observational studies in epidemiology (STROBE) guidelines.

### 2.2. Microbiological Data

Results from all microbiological samples were evaluated; the presence of MDR resistance was defined as the identification of a MDR pathogen present in any microbiological specimen within 15 days of the TDM measurement. A MDR pathogen was defined using the European Centre for Disease Control (ECDC) and Centre for Disease Control and Prevention (CDC) criteria [21]. In patients previously colonized with MDR pathogens, we considered development of a new MDR colonization or infection to have occurred if the mechanism of resistance of the identified strain was different from the one previously detected. In our ICU, routine surveillance cultures (i.e., rectal swabs, nasal swabs, tracheal aspirates and urinary cultures) were sampled on admission and twice a week thereafter throughout the entire ICU stay. Nasal swabs were specifically directed to the detection of methicillin-resistant *Staphyloccocus aureus* (MRSA), using ChromID^®^ MRSA selective plates. Rectal swabs were streaked onto selective plates, in particular chromID^®^ CARBA SMART agar (BioMérieux, Craponne, France) for the detection of carbapenemase-producing *Enterobacteriaceae*, MacConkey agar containing ceftazidime (BioTRADING, Mijdrecht, The Netherlands) for the detection of third-generation cephalosporin-resistant *Pseudomonas aeruginosa*, *Klebsiella* spp. and *Enterobacter* spp. and chromID^®^ VRE agar for the detection of vancomycin-resistant *Enterococcus faecium* (VRE). Identification of MDR bacteria was performed using matrix-assisted laser desorption/ionization time-of-flight analysis (MALDI–TOF).

Drug resistance was defined according to breakpoints recommended by the European Committee on Antimicrobial Susceptibility Testing (EUCAST) [22], using the disk diffusion method. Carbapenemases (i.e., OXA-48, KPC, NDM, VIM and IMP) were detected via Polymerase Chain Reaction (PCR) analysis or Coris Resist-5 O.O.K.N.V. antigenic detection (Coris BioConcept, Isnes, Belgium). VanA and VanB genes were detected for VRE strains via PCR analysis. ESBL-producing *Enterobacteriaceae* and AmpC de-repression were identified using detection of synergy on the disk diffusion test as recommended by EUCAST. MDR *Pseudomonas* and *Acinetobacter* spp. were defined as recommended, considering antimicrobial resistance phenotypes [21].

### 2.3. Therapeutic Drug Monitoring

TDM of broad-spectrum β-lactams has been routinely performed in our ICU since October 2009. β-lactam concentrations were measured on one blood sample of 3 mL, just before (T0 or C_min_) drug administration [23]. The exact time of sampling was recorded. Samples were kept on ice and sent directly to the clinical chemistry laboratory; after centrifugation at 3000 rpm at 4 °C for 10 min, the supernatant was removed and analyzed. The serum concentrations of the four β-lactams (ceftazidime, cefepime, piperacillin/tazobactam and meropenem) were determined using high-performance liquid chromatography connected to UV spectro-photometry.

For every TDM, we assessed whether C_min_ was higher than the clinical breakpoint for *Pseudomonas aeruginosa* as defined by the EUCAST, i.e., 8 mg/L for cefepime/ceftazidime (CEF), 16 mg/L for piperacillin/tazobactam (TZP) and 2 mg/L for meropenem (MEM). This threshold was selected by targeting the less susceptible pathogen for each drug and according to recent recommendations [24].

As the minimum antibiotic concentration to achieve antimicrobial suppression is higher than the MIC for microbiological eradication and clinical cure [7,15], we also calculated a more conservative drug threshold (i.e., sensitivity analysis), corresponding to C_min_ exceeding 4 times the clinical breakpoint of *Pseudomonas aeruginosa* as previously reported for beta-lactams [7]. Thus, target drug concentrations were 32 mg/L for CEF, 64 mg/L for TZP and 8 mg/L for MEM. Drug concentrations below these levels were considered “insufficient”.

For each patient, we considered the lowest drug concentrations (C_min_) for the final analysis in the event of multiple TDMs.

### 2.4. Data Collection

We collected demographic data (age, gender, ethnicity, weight), co-morbidities (immunosuppression, chronic heart failure, arterial hypertension, diabetes, chronic obstructive pulmonary disease (COPD) or asthma, liver cirrhosis or chronic kidney disease) and the use of supportive care during the ICU stay (i.e., vasopressors, mechanical ventilation, renal replacement therapy, extracorporeal membrane oxygenation, ECMO). The antibiotic regimen on the days of TDM was also recorded. We collected data on known risk factors for MDR, such as intravenous catheters (from admission to TDM day), urinary tract catheters (from admission to TDM day), previous hospitalization (i.e., within the 6 months preceding the actual admission), previous antibiotic use (i.e., within the 3 months preceding the actual admission), any surgical intervention (i.e., within the 3 months preceding the actual admission) and previous colonization/infection by MDR pathogens (i.e., within the 3 months preceding the actual admission).

The severity of the disease was assessed using the Acute Physiology and Chronic Health Evaluation (APACHE) II score [25] on admission and the Sequential Organ Failure Assessment (SOFA) score [26] on the day of admission and the day of the first TDM for each patient.

Renal function was estimated using creatinine clearance (CrCL), which was calculated using the 24 h urine collection of the day before the TDM and the creatinine value of the TDM day, with the following formula: CrCL (mL/min) = [(24-h urine volume, mL) × urinary creatinine concentration, mg/dL)/(serum creatinine concentration, mg/dL × 1440 min)].

Hospital mortality was also collected and refers to any death occurring in the ICU or during hospital stay after ICU discharge.

### 2.5. Study Outcomes

The primary outcome of this study was the acquisition of MDR pathogens within 15 days of the TDM and its association with the presence of insufficient drug concentrations. Secondary outcomes included the occurrence of insufficient antibiotic levels among different classes of antibiotics.

### 2.6. Statistical Analysis

Statistical analyses were performed using SPSS for MacIntosh NT v.27.0 (SPSS Inc., Chicago, IL, USA) and STATA SE17. Descriptive statistics were calculated for all study variables. A Kolmogorov–Smirnov test was used, and histograms and normal quartile plots were examined to verify the normality of distribution of continuous variables. Discrete variables were expressed as counts (%) and continuous variables as mean ± standard deviation or median (interquartile range), as appropriate. Demographics and clinical differences between study groups were assessed using a Chi-square test, Fisher’s exact test, Student’s *t*-test or a Mann–Whitney U-test, as appropriate.

A competing risk analysis and graph was performed to assess the association of insufficient drug levels and MDR, the competing event being death within 15 days of TDM. Time to event was calculated as the day of MDR pathogen identification minus the day of TDM. If no MDR pathogen was identified, time to event was calculated as day of death/discharge minus TDM (if less than 15 days). Sub-hazard ratios (sHR) and 95% confidence intervals (CI) were computed for all variables included in the model. We adjusted the model for known variables associated with AMR development. A receiver operator curve (ROC) was constructed and the area under the ROC (AUROC) was calculated to analyze the ability of the ratio between C_min_ and the clinical breakpoint of *Pseudomonas aeruginosa* to predict the acquisition of MDR pathogens. A *p*-value of <0.05 was considered statistically significant.

## 3. Results

### 3.1. Study Population

We identified 548 eligible patients, of whom 444 fulfilled the inclusion criteria (male sex, *n* = 313, 71%; female sex, *n* = 131, 29%; mean age 58 ± 15 years; Table 1). The median time between admission and antibiotic administration was 1 (0–5) day, and about half of patients required mechanical ventilation (234/444, 53%) and vasopressors (23, 52%) during the ICU stay. A total of 845 TDMs were performed; the median time between antibiotic initiation and the first TDM was 2 (1–4) days. Hospital mortality occurred in 191 (43%) patients. The most common infection was pneumonia (222/444, 50%) followed by intra-abdominal infection (84/444, 19%) (Supplementary Table S1).

### 3.2. Insufficient Drug Levels (Less than 100%fT > 1xMIC)

A total of 65 (15%) patients had insufficient drug levels: 49/168 (29%) patients had insufficient TZP levels (C_min_ = 4.4 [2.5–8.2] mg/L), 9/218 (4%) patients had insufficient MEM levels (C_min_ = 1.4 [1.0–1.9] mg/L) and 7/58 (12%) patients had insufficient CEF levels (*p* < 0.001–3/17, [18%] patients had insufficient cefepime levels, C_min_ = 2.3 [2.2–4.3] mg/L and 4/41 [10%] patients had insufficient ceftazidime levels, C_min_ = 5.5 [2.8–6.0] mg/L).

### 3.3. MDR Acquisition

**Figure 1:**
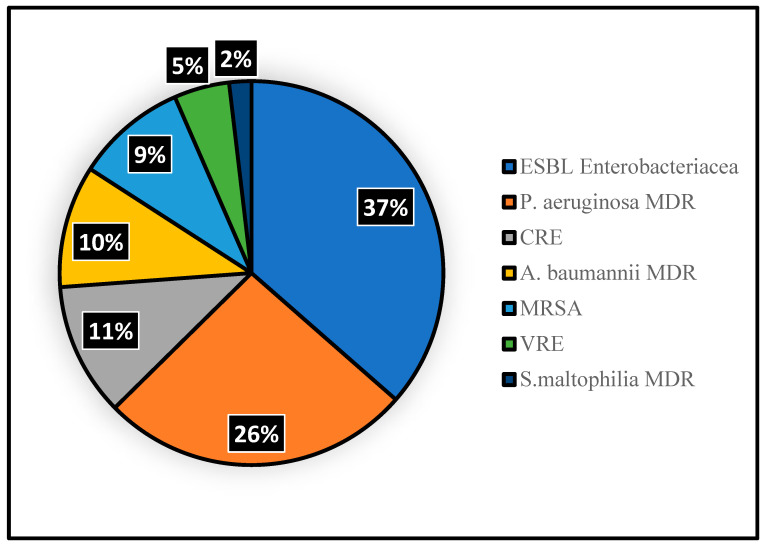
Distribution of multi-drug resistant (MDR) bacteria acquisition in the study cohort. A total of 110 different resistant pathogens were identified in 93 patients. Extended Spectrum Beta-Lactamase (ESBL) *Enterobacteriacea* (*N* = 39): *Citrobacter freundii* (*N* = 4), *Citrobacter amalonati* (*n* = 1), *Enterobacter aerogenes* (*n* = 2), *Enterobacter cloacae* (*N* = 5), *Escherichia coli* (*N* = 19), *Klebsiella oxytoca* (*N* = 3), *Klebsiella pneumoniae* (*N* = 5). MDR *Pseudomonas aeruginosa* (*N* = 28 of which 7 were VIM and 2 IMP producers). Carbapenem Resistant *Enterobacteriacea* (CRE, *N* = 12): *Serratia marcescens* (*n* = 1), *Enterobacter cloacae* (*N* = 2; 1 OXA-48 and 1 VIM), *Escherichia coli* (*N* = 1; KPC), *Klebsiella oxytoca* (*N* = 2; 1 KPC and 1 OXA-48), *Klebsiella pneumoniae* (*N* = 6; 5 KPC and 1 OXA-48). MDR *Acinetobacter baumannii* (*N* = 11 of which 2 were OXA-23 producers). Methicillin Resistant *Staphylococcus aureus* (MRSA, *N* = 10). MDR *Stenotrophomonas maltophilia* (*N* = 2).

A total of 93 (21%) patients acquired 110 MDR bacteria within 15 days of TDM. The most identified MDR pathogens were *Pseudomonas aeruginosa* (28/110; 26%) and *Escherichia coli* (19/110; 17%), as shown in Figure 1.

The characteristics of patients according to the acquisition of MDR bacteria are reported in Table 1. The group that acquired MDR pathogens had a higher prevalence of female patients and a higher incidence of previous colonization by other MDR pathogens with different resistance mechanisms from the ones identified during the study, when compared to others.

### 3.4. Insufficient Drug Levels (Less than 100%fT > 1xMIC) and MDR Acquisition

**Figure 2:**
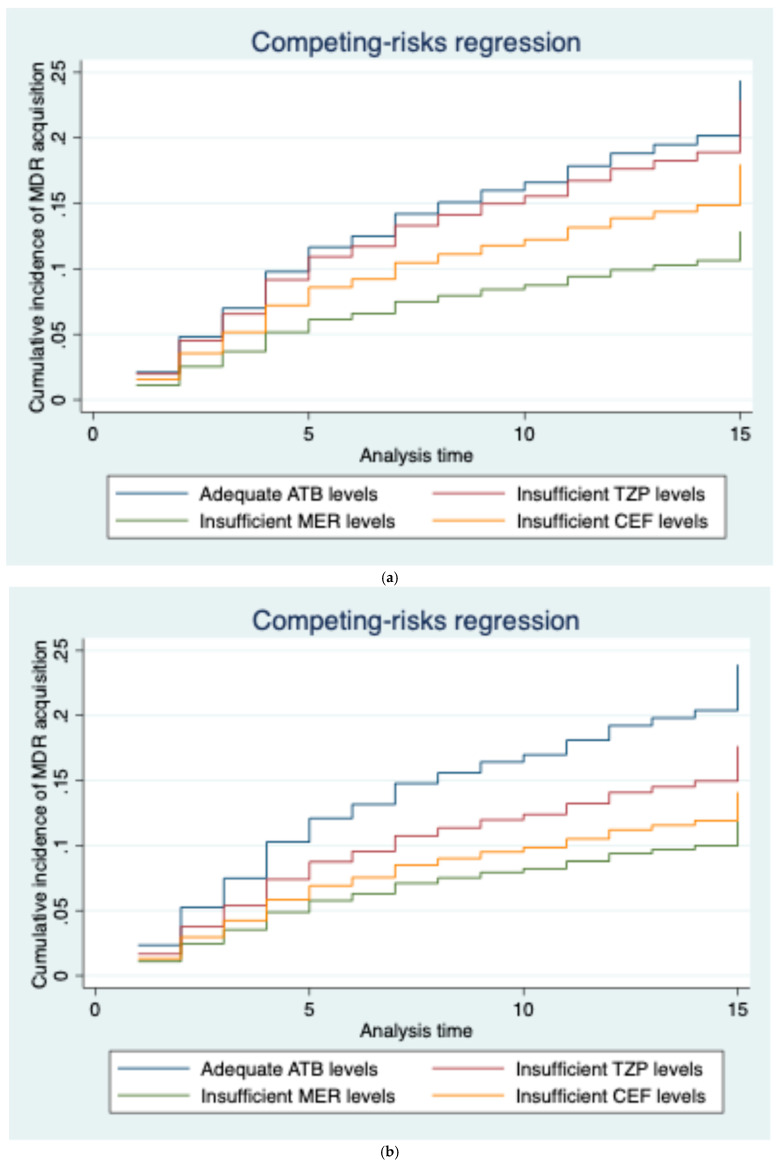
Cumulative incidence of multi-drug-resistant pathogen acquisition over time according to serum antibiotic levels. Panel (**a**) Insufficient antibiotic levels were defined as less than 100% fT > MIC; *p* = 0.62. Panel (**b**) Insufficient antibiotic levels were defined as less than 100% > 4xMIC (the minimum inhibitory concentration for resistance suppression in beta-lactams); *p* = 0.08. *p*-values were calculated using a multivariable competing risk model. ATB: antibiotic; MER: meropenem; PIP: piperacillin–tazobactam; CEF: cefepime/ceftazidime.

The proportion of patients with a new MDR acquisition was not different between patients with insufficient and adequate drug levels (9/65, 14% vs. 84/379, 22%; OR 0.56 [95%CI 0.27–1.19]—*p* = 0.13). Also, the time from the first TDM to the acquisition of MDR was similar between groups (13 [8–22] vs. 12 [8–19] days; *p* = 0.40). Similarly, patients with and without MDR acquisition had a similar proportion of insufficient antibiotic concentrations (9/93, 10% in the MDR group vs. 56/351, 16% in the other, *p* = 0.14). The characteristics of patients according to drug levels are shown in Supplementary Table S2. In the multivariable competing-risk model analysis, male gender was the only variable independently associated with a decreased probability of MDR acquisition (Table 2); insufficient drug level (sHR 0.84 [95% CI 0.42–1.68]) was not associated with MDR bacteria acquisition (Figure 2a). The AUROC curve of the ratio between C_min_ and the clinical breakpoint of *Pseudomonas aeruginosa* to predict AMR acquisition was 0.49 (95% CI 0.42–0.55).

**Figure 3:**
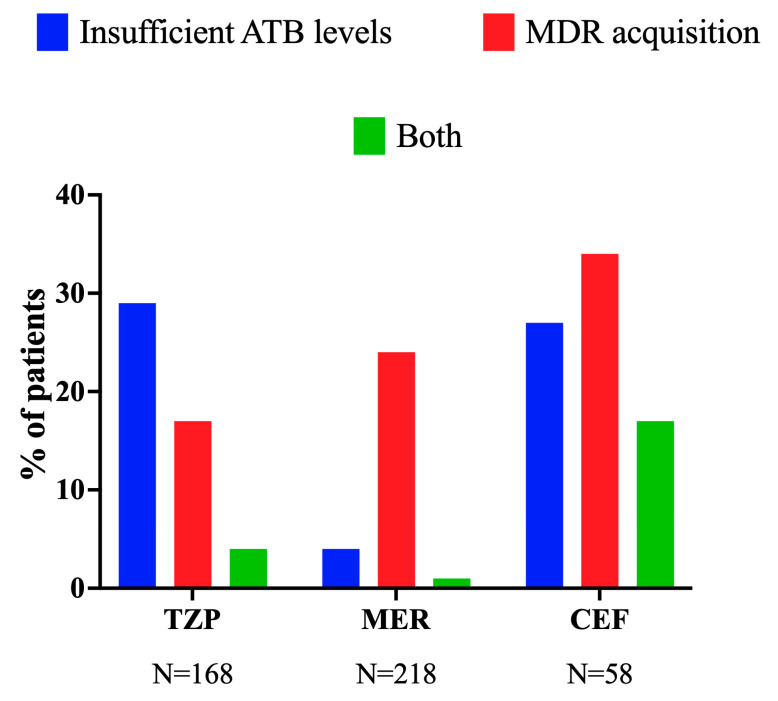
Incidence of antimicrobial-resistant and insufficient antibiotic levels according to different beta-lactams. Insufficient antibiotic levels were defined as less than 100% > MIC. MER: meropenem; PIP: piperacillin–tazobactam; CEF: ceftazidime/cefepime. The group “both” refers to the presence of MDR in patients who had insufficient antibiotic levels.

Among the 168 patients treated with TZP, 29 (17%) acquired a MDR bacteria, with 7 of those (24%) having insufficient drug concentrations (sHR 0.71 [95% CI 0.33–1.55]). Among the 218 patients treated with MEM, 53 (24%) acquired a MDR bacteria, with 1 of those (2%) having insufficient drug concentrations (sHR 0.46 [95% CI 0.06–3.66]). Among the 58 patients treated with CEF, 11 (19%) acquired a MDR bacteria, with 1/11 of them (9%) having insufficient drug concentrations (sHR 0.56 [0.09–3.63]; 2/17 patients with cefepime, 0 with insufficient drug concentrations; 9/41 with ceftazidime, 1 with insufficient drug concentrations—Figure 3).

### 3.5. Sensitivity Analysis

Using the more conservative antibiotic definition of insufficient drug levels (T0 or C_min_ < 4xMIC), which is the reported minimum inhibitory concentration for resistance suppression described for beta-lactams, 305 (69%) patients had insufficient antibiotic concentrations. According to this definition, the characteristic of patients with insufficient antibiotic levels is shown in Supplementary Table S3. In a multivariable competing-risk analysis (Table 2), insufficient drug levels (sHR 1.52 [95% CI 0.95–2.41]) were not associated with MDR bacteria acquisition (Figure 2b).

One and twenty two treated (73%) of patients treated with TZP had insufficient antibiotic levels; 152/218 (70%) patients treated with MEM had insufficient antibiotic level and 31/58 (53%) patients treated with CEF had insufficient antibiotic levels (10/17, 59% of those treated with cefepime and 21/41, 51% of those treated with ceftazidime).

Among the 168 patients treated with TZP, 29 (17%) acquired a MDR bacteria, with 19 of them (66%) having insufficient drug concentrations. Among the 218 patients treated with MEM, 53 (24%) acquired a MDR bacteria, with 40 of them (76%) having insufficient drug concentrations. Among the 58 patients treated with CEF, 11 (34%) acquired a MDR bacteria, with 7/11 of them (64%) having insufficient drug concentrations (2/17 patients with cefepime, 2 with insufficient drug concentrations; 9/41 with ceftazidime, 5 with insufficient drug concentrations).

## 4. Discussion

In this retrospective cohort of critically ill patients treated with beta-lactam antibiotics, no association between insufficient drug levels and the acquisition of AMR within 15 days was found. Also, only 15% of patients had insufficient antibiotic levels, when recently published definitions were implemented.

Antibiotic therapy is one of the main drivers of antimicrobial resistance [27]. Optimizing antimicrobial therapy can potentially overcome this phenomenon [28]; optimal antimicrobial therapy would therefore aim to limit or minimize the risk of death related to infection, prevent complications and reduce the length of the illness while limiting potential side effects related to antimicrobial use, such as toxicity, allergy, microbiome disruption and AMR [29]. When optimizing antimicrobial use, physicians should focus on adequately choosing which antibiotic to use based on the clinical setting (i.e., community-acquired vs. hospital-acquired pathogens) and known local resistance patterns, avoiding delay in starting therapy and ensuring correct antibiotic dosing based on the patient’s characteristics and site/type of infection [11]. In this context, clinically ill patients are highly susceptible to antibiotic underdosing [30], mainly due to increased volume of distribution and augmented renal clearance. In fact, augmented renal clearance has been described in sepsis, polytrauma and burn victims, traumatic brain injury and subarachnoid hemorrhage, ventilator-associated pneumonia and post-operative patients [31,32,33,34,35,36,37]. Interestingly, augmented clinical clearance has been associated with sub-therapeutic beta-lactam concentrations not only due to increased renal drug elimination [38], but also related to through concentrations [39,40].

In the ICU setting, 16 to 44% of patients have previously been shown to have adequate antibiotic serum levels [23]. In our cohort, we found, depending on the threshold, a rate of 31 to 85% of adequate antibiotic levels. Regardless of the threshold used for patients, insufficient drug levels did not increase the risk of AMR. This is in contrast with a previous study showing that lower concentrations given prophylactically after lung transplantation were associated with the development of infectious complications, such as AMR and infection, in these patients [41]. How to explain these findings? First, monitoring drug levels may have led to adjusting rapidly drug regimens, resulting in adequate drug concentrations. Second, we evaluated a very heterogeneous population of critically ill patients, while the previous study assessed AMR in immunosuppressed patients, in whom the role of the antibiotic, in the absence of a normal immune response, could be more relevant than in other settings. Third, MDR acquisitions could also be influenced by other confounders (i.e., intravascular devices, hand hygiene, isolation policies), which would be independent of antibiotic concentrations. Fourth, only one TDM with different sampling time was used for each patient, and AMR included other bacterial species; as such, the drug exposure required to suppress the emergence of resistance, the duration of therapy and the specific susceptibility of each isolate could have blunted an adequate evaluation of the study outcome [7].

Several studies have suggested that for beta-lactams, the PK/PD target for promoting clinical cure [19,42,43,44,45] is lower than the one for suppressing resistance [7,46,47,48]. Since antibiotic resistance leads to a greater economic burden and higher mortality and morbidity, with a negative impact on healthcare systems [49], it would be reasonable to suggest that clinicians target the higher C_min_ targets possible for beta-lactams in this setting [7]. However, in our study, even the higher threshold to define insufficient drug concentrations was not associated with the emergence of AMR. This may happen because for any antibiotic, even if used optimally, selection pressure and resistance acquisition are triggered by the pathogen’s exposure to the drug [50]. Moreover, a larger initial bacterial burden has a higher probability of having a first-step mutant susceptible to selection during antibiotic use and leading to regrowth and resistance spread [51]. In this setting the concept of the mutant prevention concentration (MPC) becomes relevant. The MPC is the concentration that prevents the growth of first-step resistance [52]. In order to suppress resistance, antibiotic concentrations should be above the MPC and not only the MIC. In fact, antibiotic concentrations that are higher than the MIC but below the MCP (mutant selection window) have the higher potential of selecting resistant first-step mutants and promoting regrowth and AMR [53,54]. Therefore, using MIC as a target for resistance suppression may not be appropriate [55] as a recent study has shown that, depending on the pathogen and the strain, MPC can be 4 to 32 times higher than the MIC [56]. Indeed, in a retrospective study, continuous infusion to optimize beta-lactam antibiotic concentrations targeting MIC did not reduce the probability of AMR acquisition when compared to standard dose regimens [57]. In this setting, the duration of therapy and the initial inoculum size might be even more relevant parameters to observe. Additionally, Dhaese et al. [56] have introduced the concept of the highest maximum dose, which would maximize tissue concentration and optimize cell kill, minimizing toxicity. However, using very high beta-lactam regimens might also result in excessive drug concentrations, which might be associated with drug toxicity, especially neurotoxicity, and poor outcome [58,59,60].

This study has several limitations. First, we conducted a retrospective study, and we had to rely on the adequate filling of the charts for the accuracy of the information provided. This may have generated a selection bias as the decision to perform TDM was taken by the health care team and not documented for retrospective analysis. Similarly, due to a lack of a protocol approach to guide TDMs, patients had varying numbers of TDMs, with some only having one TDM performed. Therefore, we considered only the lowest TDM for each patient. However, after the initial insufficient TDM, drug regimens were adjusted, and the following TDMs in patients that had them were often adequate, which may have had an impact on successfully suppressing resistance. Second, as a single-center study, our results may not apply to other centers with different hygiene and isolation policies. Moreover, we were unable to assess adherence to infection control measures retrospectively and how this may have impacted our results. It should be noted that, during the study period (2009 to 2014), antimicrobial stewardship had not been consistently implemented in our center. Third, we assessed AMR within 15 days of TDM and not the initiation of antibiotic therapy; however, as the lowest TDM occurred early, this would have only marginally influenced our findings. Fourth, we did not separate patients who became colonized from those who became infected with MDR bacteria, which has a significant impact on clinical outcomes such as mortality. However, our primary endpoint was to investigate AMR development and not clinical outcomes. Fifth, we did not assess the cause of the low TDM for each patient, which may impact the development of resistance differently. Finally, we only focused on MDR strains, while it is possible that other forms (i.e., more limited) of resistance might have emerged during antibiotic therapy; however, MDR treatment remains the main challenge for ICU physicians and was considered a more relevant target to be evaluated in this study.

## 5. Conclusions

In our cohort, insufficient antibiotic levels were not associated with acquiring MDR bacteria. This was also the case when considering the previously described minimum inhibitory concentration for resistance suppression in beta-lactams. Future studies should focus on identifying the mutant prevention concentration of each isolated pathogen and on comparing antimicrobial therapy targeting MIC and antimicrobial therapy targeting MPC to assess development of MDR bacteria, clinical cure, toxicity and cost effectiveness of using a higher beta-lactam dose.

## Figures and Tables

**Table 1:** Characteristics of the study population, according to the acquisition of antimicrobial resistance (AMR). Data are presented as count (percentage), mean (SD) or median (interquartile rage).

	ALL (*N* = 444)	No AMR (*N* = 351)	AMR Acquisition (*N* = 93)	*p*-Value
Male sex, *n* (%)	313 (71)	258 (74)	55 (59)	0.01
Age, mean (SD)	58 (±15)	57 (±15)	56 (±14)	0.14
APACHE II score on admission, median (IQR)	23 (17–29)	23 (17–29)	25 (17–31)	0.20
SOFA score, median (IQR)	10 (7–14)	10 (7–14)	10 (7–14)	0.88
Arterial Hypertension, *n* (%)	184 (41)	151 (43)	33 (36)	0.20
Heart disease, *n* (%)	221 (50)	176 (50)	45 (48)	0.83
Diabetes Mellitus, *n* (%)	113 (26)	92 (26)	21 (23)	0.51
COPD/Asthma, *n* (%)	87 (20)	72 (21)	15 (16)	0.38
Immunosuppression, *n* (%)	127 (29)	100 (29)	27 (29)	0.90
Cirrhosis, *n* (%)	33 (7)	29 (8)	4 (4)	0.27
Chronic Kidney Disease, *n* (%)	82 (19)	67 (19)	15 (16)	0.55
Chronic Renal Replacement therapy, *n* (%) *	68 (15)	54 (15)	14 (15)	0.99
Malignancy, *n* (%)	23 (5)	19 (5)	4 (4)	0.99
Time between admission and antibiotic use, days	1 (0–5)	1 (0–5)	1 (0–4)	0.19
Time between antibiotic initiation and TDM, days	2 (1–4)	2 (1–4)	2 (1–3)	0.84
Time at risk, days	7 (3–12)	7 (3–13)	5 (3–11)	0.17
During ICU stay				
Vasopressor, *n* (%)	230 (52)	180 (51)	50 (54)	0.73
Inotropes, *n* (%)	130 (29)	100 (29)	30 (32)	0.52
Mechanical ventilation, *n* (%)	234 (53)	187 (53)	47 (51)	0.64
Before MDR acquisition				
Antibiotic use in the previous 3 months, *n* (%)	181 (41)	142 (41)	39 (42)	0.81
Hospitalization in the previous 3 months, *n* (%)	202 (46)	152 (43)	50 (54)	0.08
Surgery in the previous 3 months, *n* (%)	129 (29)	96 (27)	33 (36)	0.13
Mechanical ventilation, *n* (%)	302 (68)	239 (68)	63 (68)	0.99
Central venous catheter, *n* (%)	364 (82)	289 (82)	75 (81)	0.76
Urinary tract catheter, *n* (%)	329 (74)	257 (73)	72 (77)	0.51
RRT in ICU *n*, (%) #	68 (15)	54 (15)	14 (15)	0.99
ECMO, *n* (%)	24 (5)	19 (5)	5 (5)	0.99
Previous Colonization by other drug-resistant bacteria, *n* (%)	68 (15)	67 (19)	27 (29)	0.05
Source control, *n* (%)	95 (21)	75 (21)	20 (20)	0.99
C_min_ levels < 1xMIC, *n* (%)	65 (15)	56 (16)	9 (10)	0.14
C_min_ levels < 4xMIC, *n* (%)	305 (69)	239 (68)	66 (71)	0.62
Outcomes				
ICU length of stay, days, median (IQR)	14 (10–23)	14 (9–21)	18 (12–30)	0.001
ICU mortality, *n* (%)	131 (30)	110 (31)	21 (22)	0.13
Hospital mortality, *n* (%)	191 (43)	156 (44)	35 (38)	0.29

AMR: antimicrobial resistance; APACHE: acute physiological and chronic health evaluation; SOFA: sequential organ dysfunction assessment; COPD: chronic obstructive pulmonary disease; TDM: therapeutic drug monitoring; RRT: renal replacement therapy; ECMO: extracorporeal membrane oxygenation; C_min_: minimal antibiotic concentration; MIC: minimum inhibitory concentration; ICU: intensive care unit. * Chronic renal replacement therapy refers to patients with end-stage renal disease who had received renal replacement therapy before ICU admission. # any renal replacement therapy occurring before MDR acquisition including patients in chronic RRT (as defined above) and novel RRT after ICU admission due to acute kidney injury. No patients started RRT for the first time after ICU admission and before MDR acquisition.

**Table 2:** Multivariable competing risk analysis for factors independently associated with the acquisition of antimicrobial resistance (AMR). Results are expressed as sub-hazard ratios (sHR) and 95% confidence intervals (95% CI). Model 1 = C_min_ < MIC (MIC = minimal inhibitory concentration, corresponding to the clinical breakpoint of *Pseudomonas aeruginosa* for each drug); Model 2 = C_min_ < 4xMIC (minimum inhibitory concentration for resistance suppression for beta-lactams). Characteristics of the study population, according to the acquisition of antimicrobial resistance (AMR). Data are presented as count (percentage), mean (SD) or median (interquartile rage).

Model 1	Multivariable Analysis sHR (95% CI)	Model 2	Multivariable Analysis sHR (95% CI)
Insufficient antibiotic levels	0.84 (0.42–1.68)	Insufficient antibiotic levels	1.52 (0.95–2.41)
Male gender	0.57 (0.38–0.88)	Male gender	0.55 (0.36–0.83)
APACHE score on admission	1.02 (0.99–1.05)	APACHE score on admission	1.03 (0.99–1.06)
Previous colonization by other MDR bacteria	1.05 (0.97–2.33)	Previous colonization by other MDR bacteria	1.48 (0.96–2.29)
Urinary tract catheter	0.92 (0.54–1.58)	Urinary tract catheter	0.93 (0.53–1.61)
Central venous catheter	0.86 (0.57–1.45)	Central venous catheter	0.81 (0.45–1.47)
Mechanical ventilation	0.91 (0.57–1.45)	Mechanical ventilation	0.90 (0.57–1.45)
Previous hospitalization	1.27 (0.84–1.92)	Previous hospitalization	1.48 (0.96–2.29)
Previous surgery	1.35 (0.87–2.09)	Previous surgery	1.39 (0.90–2.15)

MDR: multi-drug-resistant; sHR: sub-hazard ratio.

## Data Availability

All data generated or analyzed during this study are included in this published article and its Supplementary Information files.

## Supplementary Materials

The following supporting information can be downloaded at: https://www.mdpi.com/article/10.3390/life15050739/s1, Table S1: Type of infection; Table S2: Characteristics of the study population, according to the results of therapeutic drug monitoring (insufficient vs. adequate). Data are presented as counts (%), mean ± standard deviation or median (IQRs); Table S3: Characteristics of the studied population according to beta-lactams levels, using a higher threshold to define insufficient antibiotic levels (i.e., C_min_ < 4xMIC).

## Abbreviations

The following abbreviations are used in this manuscript:

MDR	Multi-drug resistance
AMR	Antimicrobial resistance
TDM	Therapeutic drug monitoring
MPC	Mutant prevention concentration
MIC	Minimum inhibitory concentration
Pk/Pd	Pharmacokinetic/pharmacodynamic
CEF	Ceftazidime/Cefepime
TZP	Tazobactam–piperacillin
MEM	Meropenem
ICU	Intensive Care Unit
APACHE	acute physiological and chronic health evaluation
SOFA	sequential organ dysfunction assessment
COPD	chronic obstructive pulmonary disease
ECMO	extracorporeal membrane oxygenation
RRT	renal replacement therapy

## References

[1] Antimicrobial Resistence. https://www.who.int/news-room/fact-sheets/detail/antimicrobial-resistance.

[2] Antimicrobial Resistance Collaborators (2022). Global burden of bacterial antimicrobial resistance in 2019: A systematic analysis. Lancet.

[3] Luepke K.H., Mohr J.F. (2017). The antibiotic pipeline: Reviving research and development and speeding drugs to market. Expert. Rev. Anti Infect. Ther..

[4] Wheatley R., Diaz Caballero J., Kapel N., de Winter F.H.R., Jangir P., Quinn A., Del Barrio-Tofino E., Lopez-Causape C., Hedge J., Torrens G. (2021). Rapid evolution and host immunity drive the rise and fall of carbapenem resistance during an acute *Pseudomonas aeruginosa* infection. Nat. Commun..

[5] Cabot G., Bruchmann S., Mulet X., Zamorano L., Moya B., Juan C., Haussler S., Oliver A. (2014). *Pseudomonas aeruginosa* ceftolozane-tazobactam resistance development requires multiple mutations leading to overexpression and structural modification of AmpC. Antimicrob. Agents Chemother..

[6] Shields R.K., Chen L., Cheng S., Chavda K.D., Press E.G., Snyder A., Pandey R., Doi Y., Kreiswirth B.N., Nguyen M.H. (2017). Emergence of Ceftazidime-Avibactam Resistance Due to Plasmid-Borne blaKPC-3 Mutations during Treatment of Carbapenem-Resistant *Klebsiella pneumoniae* Infections. Antimicrob. Agents Chemother..

[7] Sumi C.D., Heffernan A.J., Lipman J., Roberts J.A., Sime F.B. (2019). What Antibiotic Exposures Are Required to Suppress the Emergence of Resistance for Gram-Negative Bacteria? A Systematic Review. Clin. Pharmacokinet..

[8] Vincent J.L., Sakr Y., Singer M., Martin-Loeches I., Machado F.R., Marshall J.C., Finfer S., Pelosi P., Brazzi L., Aditianingsih D. (2020). Prevalence and Outcomes of Infection Among Patients in Intensive Care Units in 2017. JAMA.

[9] Canton R., Akova M., Carmeli Y., Giske C.G., Glupczynski Y., Gniadkowski M., Livermore D.M., Miriagou V., Naas T., Rossolini G.M. (2012). Rapid evolution and spread of carbapenemases among *Enterobacteriaceae* in Europe. Clin. Microbiol. Infect..

[10] Brusselaers N., Vogelaers D., Blot S. (2011). The rising problem of antimicrobial resistance in the intensive care unit. Ann. Intensive Care.

[11] Martinez M.N., Papich M.G., Drusano G.L. (2012). Dosing regimen matters: The importance of early intervention and rapid attainment of the pharmacokinetic/pharmacodynamic target. Antimicrob. Agents Chemother..

[12] Pinder M., Bellomo R., Lipman J. (2002). Pharmacological principles of antibiotic prescription in the critically ill. Anaesth. Intensive Care.

[13] Bloos F., Ruddel H., Thomas-Ruddel D., Schwarzkopf D., Pausch C., Harbarth S., Schreiber T., Grundling M., Marshall J., Simon P. (2017). Effect of a multifaceted educational intervention for anti-infectious measures on sepsis mortality: A cluster randomized trial. Intensive Care Med..

[14] Li J., Xie S., Ahmed S., Wang F., Gu Y., Zhang C., Chai X., Wu Y., Cai J., Cheng G. (2017). Antimicrobial Activity and Resistance: Influencing Factors. Front. Pharmacol..

[15] Guilhaumou R., Benaboud S., Bennis Y., Dahyot-Fizelier C., Dailly E., Gandia P., Goutelle S., Lefeuvre S., Mongardon N., Roger C. (2019). Optimization of the treatment with beta-lactam antibiotics in critically ill patients—Guidelines from the French Society of Pharmacology and Therapeutics (Société Française de Pharmacologie et Thérapeutique—SFPT) and the French Society of Anaesthesia and Intensive Care Medicine (Société Française d’Anesthésie et Réanimation—SFAR). Crit. Care.

[16] Goncalves-Pereira J., Povoa P. (2011). Antibiotics in critically ill patients: A systematic review of the pharmacokinetics of beta-lactams. Crit. Care.

[17] Lodise T.P., Lomaestro B.M., Drusano G.L., Society of Infectious Diseases Pharmacists (2006). Application of antimicrobial pharmacodynamic concepts into clinical practice: Focus on beta-lactam antibiotics: Insights from the Society of Infectious Diseases Pharmacists. Pharmacotherapy.

[18] Roberts J.A., Lipman J. (2009). Pharmacokinetic issues for antibiotics in the critically ill patient. Crit. Care Med..

[19] Sinnollareddy M.G., Roberts M.S., Lipman J., Roberts J.A. (2012). Beta-lactam pharmacokinetics and pharmacodynamics in critically ill patients and strategies for dose optimization: A structured review. Clin. Exp. Pharmacol. Physiol..

[20] Veiga R.P., Paiva J.A. (2018). Pharmacokinetics-pharmacodynamics issues relevant for the clinical use of beta-lactam antibiotics in critically ill patients. Crit. Care.

[21] Magiorakos A.P., Srinivasan A., Carey R.B., Carmeli Y., Falagas M.E., Giske C.G., Harbarth S., Hindler J.F., Kahlmeter G., Olsson-Liljequist B. (2012). Multidrug-resistant, extensively drug-resistant and pandrug-resistant bacteria: An international expert proposal for interim standard definitions for acquired resistance. Clin. Microbiol. Infect..

[22] The European Committee on Antimicrobial Susceptibility Testing (2022). Breakpoint Tables for Interpretation of MICs and Zone Diameters, Version 12.0. https://www.eucast.org/clinical_breakpoints.

[23] Taccone F.S., Laterre P.F., Dugernier T., Spapen H., Delattre I., Wittebole X., De Backer D., Layeux B., Wallemacq P., Vincent J.L. (2010). Insufficient beta-lactam concentrations in the early phase of severe sepsis and septic shock. Crit. Care.

[24] Abdul-Aziz M.H., Alffenaar J.C., Bassetti M., Bracht H., Dimopoulos G., Marriott D., Neely M.N., Paiva J.A., Pea F., Sjovall F. (2020). Antimicrobial therapeutic drug monitoring in critically ill adult patients: A Position Paper. Intensive Care Med..

[25] Knaus W.A., Draper E.A., Wagner D.P., Zimmerman J.E. (1985). APACHE II: A severity of disease classification system. Crit. Care Med..

[26] Vincent J.L., Moreno R., Takala J., Willatts S., De Mendonca A., Bruining H., Reinhart C.K., Suter P.M., Thijs L.G. (1996). The SOFA (Sepsis-related Organ Failure Assessment) score to describe organ dysfunction/failure. On behalf of the Working Group on Sepsis-Related Problems of the European Society of Intensive Care Medicine. Intensive Care Med..

[27] Prestinaci F., Pezzotti P., Pantosti A. (2015). Antimicrobial resistance: A global multifaceted phenomenon. Pathog. Glob. Health.

[28] Vitrat V., Hautefeuille S., Janssen C., Bougon D., Sirodot M., Pagani L. (2014). Optimizing antimicrobial therapy in critically ill patients. Infect. Drug Resist..

[29] Depuydt P., De Waele J.J. (2019). Optimal and responsible use of antibiotics. Curr. Opin. Crit. Care.

[30] Carrie C., Legeron R., Petit L., Ollivier J., Cottenceau V., d’Houdain N., Boyer P., Lafitte M., Xuereb F., Sztark F. (2018). Higher than standard dosing regimen are needed to achieve optimal antibiotic exposure in critically ill patients with augmented renal clearance receiving piperacillin-tazobactam administered by continuous infusion. J. Crit. Care.

[31] Lipman J., Wallis S.C., Boots R.J. (2003). Cefepime versus cefpirome: The importance of creatinine clearance. Anesth. Analg..

[32] Ambrose P.G., Bhavnani S.M., Ellis-Grosse E.J., Drusano G.L. (2010). Pharmacokinetic-pharmacodynamic considerations in the design of hospital-acquired or ventilator-associated bacterial pneumonia studies: Look before you leap!. Clin. Infect. Dis..

[33] Udy A., Boots R., Senthuran S., Stuart J., Deans R., Lassig-Smith M., Lipman J. (2010). Augmented creatinine clearance in traumatic brain injury. Anesth. Analg..

[34] Conil J.M., Georges B., Fourcade O., Seguin T., Lavit M., Samii K., Houin G., Tack I., Saivin S. (2007). Assessment of renal function in clinical practice at the bedside of burn patients. Br. J. Clin. Pharmacol..

[35] Minville V., Asehnoune K., Ruiz S., Breden A., Georges B., Seguin T., Tack I., Jaafar A., Saivin S., Fourcade O. (2011). Increased creatinine clearance in polytrauma patients with normal serum creatinine: A retrospective observational study. Crit. Care.

[36] May C.C., Arora S., Parli S.E., Fraser J.F., Bastin M.T., Cook A.M. (2015). Augmented Renal Clearance in Patients with Subarachnoid Hemorrhage. Neurocrit. Care.

[37] Brown R., Babcock R., Talbert J., Gruenberg J., Czurak C., Campbell M. (1980). Renal function in critically ill postoperative patients: Sequential assessment of creatinine osmolar and free water clearance. Crit. Care Med..

[38] Udy A.A., Roberts J.A., Boots R.J., Paterson D.L., Lipman J. (2010). Augmented renal clearance: Implications for antibacterial dosing in the critically ill. Clin. Pharmacokinet..

[39] Conil J.M., Georges B., Mimoz O., Dieye E., Ruiz S., Cougot P., Samii K., Houin G., Saivin S. (2006). Influence of renal function on trough serum concentrations of piperacillin in intensive care unit patients. Intensive Care Med..

[40] Udy A.A., Varghese J.M., Altukroni M., Briscoe S., McWhinney B.C., Ungerer J.P., Lipman J., Roberts J.A. (2012). Subtherapeutic initial beta-lactam concentrations in select critically ill patients: Association between augmented renal clearance and low trough drug concentrations. Chest.

[41] Taccone F.S., Bogossian E.G., Tironi R.M., Antonucci E., Hites M., Knoop C., Etienne I., Jacobs F., Creteur J. (2021). Early beta-lactam concentrations and infectious complications after lung transplantation. Am. J. Transplant..

[42] Roberts J.A., Paul S.K., Akova M., Bassetti M., De Waele J.J., Dimopoulos G., Kaukonen K.M., Koulenti D., Martin C., Montravers P. (2014). DALI: Defining antibiotic levels in intensive care unit patients: Are current beta-lactam antibiotic doses sufficient for critically ill patients?. Clin. Infect. Dis..

[43] Hites M., Taccone F.S., Wolff F., Cotton F., Beumier M., De Backer D., Roisin S., Lorent S., Surin R., Seyler L. (2013). Case-control study of drug monitoring of beta-lactams in obese critically ill patients. Antimicrob. Agents Chemother..

[44] Craig W.A. (1998). Pharmacokinetic/pharmacodynamic parameters: Rationale for antibacterial dosing of mice and men. Clin. Infect. Dis..

[45] Tam V.H., Chang K.T., Zhou J., Ledesma K.R., Phe K., Gao S., Van Bambeke F., Sanchez-Diaz A.M., Zamorano L., Oliver A. (2017). Determining beta-lactam exposure threshold to suppress resistance development in Gram-negative bacteria. J. Antimicrob. Chemother..

[46] Tam V.H., Schilling A.N., Neshat S., Poole K., Melnick D.A., Coyle E.A. (2005). Optimization of meropenem minimum concentration/MIC ratio to suppress in vitro resistance of *Pseudomonas aeruginosa*. Antimicrob. Agents Chemother..

[47] Felton T.W., Goodwin J., O’Connor L., Sharp A., Gregson L., Livermore J., Howard S.J., Neely M.N., Hope W.W. (2013). Impact of Bolus dosing versus continuous infusion of Piperacillin and Tazobactam on the development of antimicrobial resistance in *Pseudomonas aeruginosa*. Antimicrob. Agents Chemother..

[48] Tam V.H., Schilling A.N., Poole K., Nikolaou M. (2007). Mathematical modelling response of *Pseudomonas aeruginosa* to meropenem. J. Antimicrob. Chemother..

[49] Friedman N.D., Temkin E., Carmeli Y. (2016). The negative impact of antibiotic resistance. Clin. Microbiol. Infect..

[50] Lipsitch M., Samore M.H. (2002). Antimicrobial use and antimicrobial resistance: A population perspective. Emerg. Infect. Dis..

[51] Dhaese S.A.M., Hoste E.A., De Waele J.J. (2022). Why We May Need Higher Doses of Beta-Lactam Antibiotics: Introducing the ‘Maximum Tolerable Dose’. Antibiotics.

[52] Li X., Wang L., Zhang X.J., Yang Y., Gong W.T., Xu B., Zhu Y.Q., Liu W. (2014). Evaluation of meropenem regimens suppressing emergence of resistance in Acinetobacter baumannii with human simulated exposure in an in vitro intravenous-infusion hollow-fiber infection model. Antimicrob. Agents Chemother..

[53] Drusano G.L., Hope W., MacGowan A., Louie A. (2015). Suppression of Emergence of Resistance in Pathogenic Bacteria: Keeping Our Powder Dry, Part 2. Antimicrob. Agents Chemother..

[54] Firsov A.A., Vostrov S.N., Lubenko I.Y., Drlica K., Portnoy Y.A., Zinner S.H. (2003). In vitro pharmacodynamic evaluation of the mutant selection window hypothesis using four fluoroquinolones against Staphylococcus aureus. Antimicrob. Agents Chemother..

[55] Gugel J., Dos Santos Pereira A., Pignatari A.C., Gales A.C. (2006). Beta-Lactam MICs correlate poorly with mutant prevention concentrations for clinical isolates of Acinetobacter spp. and *Pseudomonas aeruginosa*. Antimicrob. Agents Chemother..

[56] Sastre-Femenia M.À., Gomis-Font M.A., Oliver A. (2025). Mutant prevention concentrations and phenotypic and genomic profiling of first-step resistance mechanisms to classical and novel β-lactams in *Pseudomonas aeruginosa*. Antimicrob. Agents Chemother..

[57] Dhaese S.A.M., De Kezel M., Callant M., Boelens J., De Bus L., Depuydt P., De Waele J.J. (2018). Emergence of antimicrobial resistance to piperacillin/tazobactam or meropenem in the ICU: Intermittent versus continuous infusion. A retrospective cohort study. J. Crit. Care.

[58] Beumier M., Casu G.S., Hites M., Wolff F., Cotton F., Vincent J.L., Jacobs F., Taccone F.S. (2015). Elevated beta-lactam concentrations associated with neurological deterioration in ICU septic patients. Minerva Anestesiol..

[59] Roberts J.A., Joynt G.M., Lee A., Choi G., Bellomo R., Kanji S., Mudaliar M.Y., Peake S.L., Stephens D., Taccone F.S. (2021). The Effect of Renal Replacement Therapy and Antibiotic Dose on Antibiotic Concentrations in Critically Ill Patients: Data From the Multinational Sampling Antibiotics in Renal Replacement Therapy Study. Clin. Infect. Dis..

[60] Roger C., Louart B. (2021). Beta-Lactams Toxicity in the Intensive Care Unit: An Underestimated Collateral Damage?. Microorganisms.

