# Supplementary material for: Beta-Lactam Antibiotic Concentrations and the Acquisition of Multi-Drug Resistant Bacteria in Critically Ill Patients
Source: Life (Basel). 2025 May 2;15(5):739. doi: 10.3390/life15050739 (PMC12113430; doi:10.3390/life15050739)
Supplement: Supplementary file 1 [file life-15-00739-s001.zip › life-3581768-supplementary.pdf]

Supplemental Table S1: Type of infection.

	N=444
Pneumonia, n (%)	222 (50)
Primary bacteremia, n (%)	46 (10)
Urinary tract infection, n (%)	15 (3)
Surgical site infection, n (%)	33 (8)
Intra- abdominal infection, n (%)	84 (19)
Meningitis/ventriculitis, n (%)	9 (2)
Malignant ear infection, n (%)	2 (0.4)
Osteomyelitis, n (%)	2 (0.4)
Mediastinitis, n (%)	4 (0.8)
Pericarditis, n (%)	2 (0.4)
Indetermined source sepsis, n (%)	25 (6)

Supplemental Table S2: Characteristics of the study population, according to the results of therapeutic drug monitoring (insufficient vs. adequate). Data are presented as counts (%), mean ± standard deviation or median (IQRs).

	**ALL (N=444)**	**Adequate (N=379)**	**Insufficient (N=65)**	**p value**
Male gender, n (%)	313 (71)	264 (70)	49 (75)	0.38
Age, mean (SD)	58 (±15)	59 (±15)	56 (±17)	0.19
Weight (Kg), median (IQR)	73 (65-85)	73 (65-85)	73 (66-87)	0.78
APACHE II score on admission, median (IQR)	23 (17-29)	23 (17-29)	21 (15-28)	0.04
SOFA score, median (IQR)	10 (7-14)	10 (7-14)	7 (5-11)	0.001
Arterial hypertension, n (%)	184 (41)	167 (44)	17 (26)	0.007
Heart Disease, n (%)	221 (50)	196 (52)	25 (39)	0.06
Diabetes Mellitus, n (%)	113 (26)	98 (26)	15 (23)	0.76
COPD/Asthma, n (%)	87 (20)	79 (21)	3 (5)	0.0.01
Chronic Kidney Disease, n (%)	82 (19)	39 (28)	43 (14)	<0.001
Chronic Dialysis, n (%)	23 (5)	11 (13)	12 (6)	0.06
Cirrhosis, n (%)	33 (7)	31 (8)	2 (3)	0.20
Immunosuppression, n (%)	127 (29)	113 (30)	14 (22)	0.19
Time between admission and antibiotic use, days	1 (0-5)	1 (0-5)	4 (0-6)	0.007
Time between antibiotic initiation and TDM, days	2 (1-4)	2 (1-4)	2 (1-4)	0.38
Before Therapeutic drug monitoring				
CRRT n, (%)	105 (24)	103 (27)	2 (3)	0.001
ECMO, n (%)	24 (5)	22 (6)	2 (3)	0.55
CrCL (ml/min), median (IQR)	73 (39-126)	60 (36-110)	125 (98-181)	0.001
Albumin (g/dL), median (IQR)	2.6 (2.3-29)	2.5 (2.2-2.8)	2.8 (2.4-3.0)	0.004
Shock, n (%)	114 (26)	103 (27)	11 (17)	0.09
During ICU stay				
Vasopressor, n (%)	230 (52)	206 (55)	24 (37)	0.01
Inotropes, n (%)	130 (29)	119 (31)	11 (17)	0.02
Mechanical ventilation, n (%)	234 (53)	195 (52)	39 (60)	0.23
Outcomes				
Acquisition of MDR pathogens, n(%)	93 (21)	84 (22)	9 (14)	0.14
Time to resistance, days (median, IQR)	12 (8-19)	12 (8-19)	13 (8-22)	0.40
ICU length of stay, days, median (IQR)	14 (10-23)	14 (10-22)	16 (10-25)	0.63
ICU mortality, n (%)	131 (30)	117 (31)	14 (22)	0.14
Hospital mortality, n (%)	191 (43)	170 (45)	21 (32)	0.07

APACHE: acute physiology and chronic health evaluation; SD: standard deviation; IQR: interquartile range; SOFA: sequential organ failure assessment; CRRT: continuous renal replacement therapy; ECMO: extracorporeal membrane oxygenation; CrCL: creatinine clearance; MDR: multi-drug resistant; ICU: intensive care unit.

Supplemental Table S3: Characteristics of the studied population according to beta-lactams levels, using a higher threshold to define insufficient antibiotic levels (i.e. C_{min} < 4x MIC).

	**Adequate (N=139)**	**Insufficient (N=305)**	**p value**
Male gender, n (%)	89 (64)	244 (73)	0.06
Age, mean (SD)	60 (±14)	57 (±16)	0.06
Weight (Kg), median (IQR)	73 (67-85)	73 (65-85)	0.66
APACHE II score on admission, median (IQR)	25 (19-31)	22 (16-28)	0.001
SOFA score, median (IQR)	12 (9-16)	10 (6-13)	0.001
Arterial hypertension, n(%)	62 (45)	122 (40)	0.41
Heart disease, n(%)	72 (52)	149 (49)	0.61
Diabetes Mellitus, n(%)	38 (27)	75 (25)	0.56
COPD/Asthma, n(%)	27 (19)	60 (20)	0.53
Chronic Kidney Disease, n(%)	39 (28)	43 (14)	<0.001
Chronic Renal Replacement therapy, n (%)	11 (13)	12 (6)	0.06
Cirrhosis, n (%)	16 (12)	17 (6)	0.03
Malignancy, n (%)	10 (7)	40 (13)	0.16
Immunosuppression, n(%)	44 (32)	83 (27)	0.37
Time between admission and antibiotic use, days	0 (0-3)	2 (0-5)	0.003
Time between antibiotic initiation and TDM, days	3 (1-4)	2 (1-4)	0.04
Before Therapeutic drug monitoring			
CRRT n, (%)	61 (44)	44 (14)	0.001
ECMO, n (%)	6 (4)	18 (6)	0.65
Creatinine Clearance, median (IQR)	43 (29-69)	93 (49-150)	0.001
Albumin, median (IQR)	2.6 (2.2-2.8)	2.6 (2.3-2.9)	0.45
Shock, n (%)	42 (30)	72 (24)	0.16
During ICU stay			
Vasopressor, n (%)	80 (58)	150 (49)	0.13
Inotropes, n (%)	47 (34)	83 (27)	0.18
Mechanical ventilation, n (%)	72 (52)	162 (53)	0.84
Outcomes			
Acquisition of AMR	27 (19)	66 (22)	0.62
Time to resistance, days (median, IQR)	7 (3-14)	6 (3-12)	0.39
ICU length of stay, days, median (IQR)	15 (9-26)	14 (10-22)	0.59
ICU mortality, n (%)	49 (36)	82 (27)	0.07
Hospital mortality, n (%)	66 (48)	125 (41)	0.22

APACHE: acute physiology and chronic health evaluation; SD: standard deviation; IQR: interquartile range; SOFA: sequential organ failure assessment; CRRT: continuous renal replacement therapy; ECMO: extracorporeal membrane oxygenation; AMR: antimicrobial resistance; ICU: intensive care unit.
